# Flavonoids: nutraceutical potential for counteracting muscle atrophy

**DOI:** 10.1007/s10068-020-00816-5

**Published:** 2020-09-16

**Authors:** Changhee Kim, Jae-Kwan Hwang

**Affiliations:** Department of Biotechnology, Yonsei University, 50 Yonsei-ro, Seodaemun-gu, Seoul, 03722 Republic of Korea

**Keywords:** Flavonoids, Muscle atrophy, Mitochondrial activity, Myogenesis, Protein turnover

## Abstract

Skeletal muscle plays a vital role in the conversion of chemical energy into physical force. Muscle atrophy, characterized by a reduction in muscle mass, is a symptom of chronic disease (cachexia), aging (sarcopenia), and muscle disuse (inactivity). To date, several trials have been conducted to prevent and inhibit muscle atrophy development; however, few interventions are currently available for muscle atrophy. Recently, food ingredients, plant extracts, and phytochemicals have received attention as treatment sources to prevent muscle wasting. Flavonoids are bioactive polyphenol compounds found in foods and plants. They possess diverse biological activities, including anti-obesity, anti-diabetes, anti-cancer, anti-oxidation, and anti-inflammation. The effects of flavonoids on muscle atrophy have been investigated by monitoring molecular mechanisms involved in protein turnover, mitochondrial activity, and myogenesis. This review summarizes the reported effects of flavonoids on sarcopenia, cachexia, and disuse muscle atrophy, thus, providing an insight into the understanding of the associated molecular mechanisms.

## Introduction

Skeletal muscle, which is composed of bundles of multinucleated cells called myofibers, is the largest body organ that accounts for 40% of body weight and supports multiple body functions (Frontera and Ochala, 2015; Mukund and Subramaniam, 2020; Wells et al., 2009). First, skeletal muscle contributes to physical performance. Three different energy systems, including aerobic oxidative, anaerobic glycolytic, and phosphagen systems, are responsible for transforming chemical energy into physical force in skeletal muscle, thereby allowing humans to perform various physical activities (Frontera and Ochala, 2015; Wells et al., 2009). Secondly, along with other two organs, hepatic and adipose tissues, skeletal muscle is a representative organ to metabolize glucose, free fatty acid, and protein (Kim et al., 2019c). Therefore, skeletal muscle is a well-known target organ not only for treating metabolic diseases and symptoms, such as obesity, type 2 diabetes, hyperglycemia, and hyperlipidemia but also for maintaining body temperature (Frontera and Ochala, 2015; Kim et al., 2019c). Skeletal muscle not only stores glucoses as glycogens and catabolizes them into pyruvates for energy production (glycolysis) but also degrades fatty acids into acetyl-CoA (β-oxidation) (Wells et al., 2009). In particular, the importance of protein metabolism in skeletal muscle cannot be overlooked because skeletal muscle, which serves as a protein reservoir, stores 50–75% of the body’s protein and accounts for 30–50% of body protein turnover (Frontera and Ochala, 2015). Consequently, the relationship between protein content and muscle function has received much attention (Kim et al., 2020).

In skeletal muscle, muscle atrophy or muscle wasting is a negative symptom wherein the muscle mass, cross-sectional area, and function are reduced (Kim et al., 2020). Besides compromising muscle function, muscle atrophy reduces the efficacy of treatment, affecting the quality of life and increasing mortality and morbidity (Huang et al., 2018). Various intrinsic (e.g., genetic factors, hormone, and aging) and environmental (e.g., stress, injury, and disuse) factors lead to muscle atrophy (Lee et al., 2015; Mukund and Subramaniam, 2020). However, the main pathological conditions that contribute to muscle atrophy are chronic diseases (e.g., cancer, diabetes, obesity, AIDS, and COPD), aging, and disuse (e.g., immobilization, bed rest, and mechanical unloading) (Lee et al., 2015; Sakuma et al., 2017). Disease- and age-induced muscle atrophy are called cachexia and sarcopenia, respectively (Sakuma et al., 2017). Although the causes of sarcopenia, cachexia, and disuse muscle atrophy are different, the resulting atrophy has similar physiological characteristics, such as inflammation, oxidative stress, mitochondrial dysfunction, and protein anabolism and catabolism imbalance (Caron et al., 2011; Evans, 2010; Sakuma et al., 2017). These causes are not independent or distinct but complementary or interconnected (Bell et al., 2016; Evans, 2010). For example, disuse muscle atrophy accelerates the onset and process of sarcopenia in the elderly or cachexia in patients with cancer because of them being bedridden, hospitalized, or immobilized (Evans, 2010). Among the numerous trials that have evaluated potential therapeutics, few have demonstrated efficacy and safety, thereby limiting the clinical application of these therapeutics (Ma et al., 2018; Ziaaldini et al., 2017). Exercise training is the primary intervention for muscle atrophy (Ma et al., 2018; Yoshioka et al., 2019; Ziaaldini et al., 2017). However, because most patients with sarcopenia or cachexia are likely to be bedridden due to illness or frailness, the application of exercise training is still restricted (Evans, 2010; Frontera and Ochala, 2015). Therefore, researchers have investigated the therapeutic potential of food ingredients, plant extracts, or phytochemicals derived from food materials or medicinal plants that are easily accessible to patients with muscle atrophy (Salucci and Falcieri, 2019; Shen et al., 2019).

Flavonoids are bioactive polyphenol compounds that are ubiquitously abundant in food and plants. Currently, more than 5000 flavonoid compounds have been identified (Kawser Hossain et al., 2016). It is widely recognized that flavonoids exert anti-oxidant and anti-inflammatory properties through different mechanisms. Flavonoids can scavenge free radicals, protect against other oxidants, chelate metal ions, and increase the activity and expression of anti-oxidant enzymes (Gomes et al., 2008; Procházková et al., 2011). The anti-inflammatory effect of flavonoids not only results from their anti-oxidant activity but also depends on regulating inflammatory response-involved enzymes and signaling pathways (Gomes et al., 2008). Specifically, flavonoids inhibit inflammation-related enzymes, such as phospholipase A2, cyclooxygenase, and lipoxygenase. Additionally, flavonoids inactivate the nuclear factor kappa B (NF-kB) and mitogen-activated protein kinase (MAPK) pathways, subsequently disturbing the production of inflammatory cytokines (Chen et al., 2018a). Thus, together with anti-oxidant property, the anti-inflammatory activity of flavonoids has received attention to manage chronic inflammatory diseases (Pan et al., 2010). Flavonoids reportedly have anti-obesity, anti-diabetes, anti-cancer, and anti-osteoporotic effects (Kawser Hossain et al., 2016; Kumar and Pandey, 2013; Le Marchand, 2002; Welch and Hardcastle, 2014). Some papers have reviewed the role of flavonoids on muscle atrophy by describing the underlying molecular mechanisms (Li et al., 2020; Mukai and Terao, 2013; Salucci and Falcieri, 2019; Shen et al., 2019). However, reviews about the effect of flavonoids on muscle atrophy are still lacking. In this review, we briefly summarize the known target points for inhibiting the progression of muscle atrophy, including protein turnover, mitochondrial activity, and myogenesis. Then, we discuss the roles of representative compounds within subclasses of flavonoids on muscle atrophy by describing their potential molecular mechanisms.

## Mechanisms of actions

**Fig. 1:**
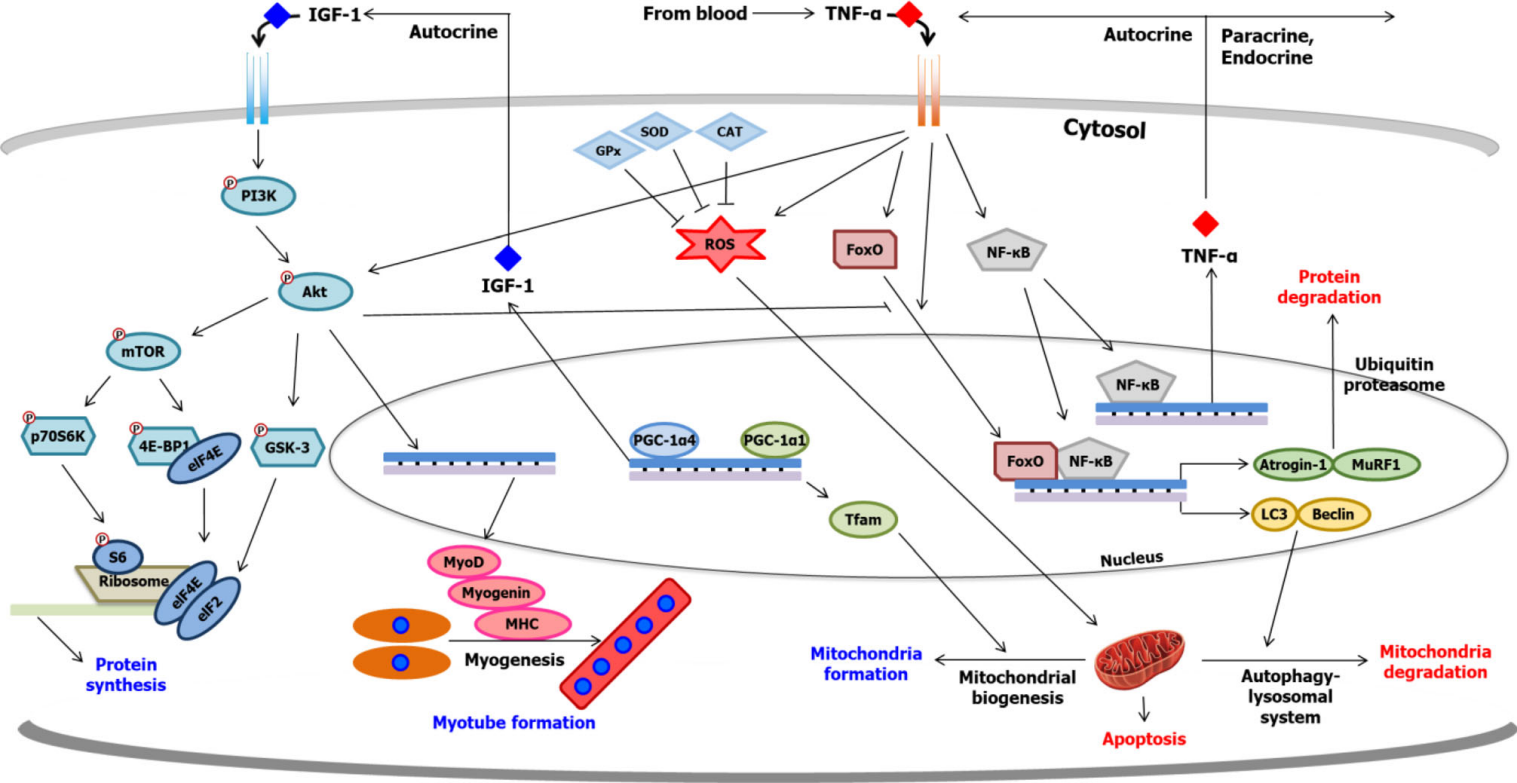
Molecular signaling pathways in skeletal muscle

Several studies have suggested that understanding the change in the signaling pathways during the development of muscle atrophy may lead to identifying and developing therapeutic agents for muscle atrophy (Kim and Hwang, 2020; Salucci and Falcieri, 2019). Three primary strategies to reverse muscle atrophy represent protein turnover, mitochondrial activity, and myogenesis. In this section, we briefly discuss each molecular pathway. Figure 1 shows a summary of the signaling pathways related to protein turnover, mitochondrial activity, and myogenesis in muscle tissue. More comprehensive reviews of these molecular and cellular mechanisms are available in existing literature (Bonaldo and Sandri, 2013; Evans, 2010; Fanzani et al., 2012; Sakuma et al., 2017).

### Protein turnover

Protein content in skeletal muscle, which is dependent on the relative rates of protein synthesis and degradation, is a major factor that determines the muscle mass (Huang et al., 2018; Phillips et al., 2005). To prevent the development of muscle atrophy and identify therapeutics to counteract it, understanding the molecular mechanisms involved in protein anabolism and catabolism is crucial. Here, we discuss the molecular signaling pathways associated with protein translation which is a critical step in protein synthesis. We also discuss the ubiquitin–proteasome system in terms of protein degradation.

The mammalian target of rapamycin (mTOR), which is a target protein of rapamycin used for immunosuppression by working as an inhibitor of mTOR (Lückemann et al., 2019), plays a key role in protein synthesis by regulating the downstream factors involved in protein translation (Kim et al., 2020). The activated mTOR propagates downstream signaling by phosphorylating the 70-kDa ribosomal S6 kinase (p70S6K) and eukaryotic initiation factor 4E binding protein 1 (4EBP-1). The ribosomal protein S6, which is a part of the mRNA translation machinery, is phosphorylated and stimulated by p70S6K (Rodriguez et al., 2014). p70S6K also phosphorylates and activates the eukaryotic initiation factor 4B (eIF4B) which regulates the 5′-cap-binding eIF4F complex (Wei et al., 2019). The phosphorylated 4EBP-1 dissociates eIF4E, which is required for the initiation of translation. The dissociated eIF4E forms a complex with eIF4G and eIF4A and binds to the 5′ cap on mRNA (Cao et al., 2019; Rodriguez et al., 2014). Consequently, the mTOR pathway promotes the translation of the mRNA into protein (Wei et al., 2019).

The ubiquitin–proteasome system is primarily responsible for protein degradation in skeletal muscle (Bonaldo and Sandri, 2013). Ubiquitin ligases play a pivotal role in stimulating muscle atrophy and directly influence a decrement in muscle mass (Yoshioka et al., 2019). In the ubiquitin–proteasome system, two ubiquitin E3 ligases, muscle atrophy F-box (MAFbx; also called atrogin-1) and muscle RING-finger protein-1 (MuRF1), are vital (Bonaldo and Sandri, 2013). Several ubiquitin molecules are attached to proteins by MuRF1 and atrogin-1, and then, the polyubiquitin-tagged protein is degraded into peptides and amino acids by 26S proteasomes (Sakuma et al., 2017). The target proteins of atrogin-1 and MuRF1 are different. Atrogin-1 attaches ubiquitin to MyoD and eIF3F (Bonaldo and Sandri, 2013; Sakuma et al., 2017), whereas MuRF1 targets sarcomeric proteins, such as troponin I, myosin heavy chain (MHC), and myosin light chain (MLC) (Bonaldo and Sandri, 2013).

Many factors or signaling pathways to regulate the protein translation and ubiquitin–proteasome system have been identified. The phosphoinositide 3-kinase (PI3K)/protein kinase B (PKB; also called Akt) pathway, which is regulated by the insulin-like growth factor 1 (IGF-1), is a primary signaling pathway for muscle hypertrophy (Egerman and Glass, 2014; Kim et al., 2020). PI3K is phosphorylated and activated by insulin receptor substrate-1 (IRS-1) when the IGF-1 binds to the IGF-1 receptor (IGF-1R) (Egerman and Glass, 2014). When PI3K stimulates Akt through phosphorylation, Akt activates the mTOR pathway for protein translation (Park et al., 2017). In addition to regulating p70S6K and 4EBP-1, Akt also participates in preventing glycogen synthase kinase-3 (GSK3), which activates translation through eIF2B (Park et al., 2017; Rommel et al., 2001). In addition, Akt inhibits forkhead box O (FoxO), a transcription factor that regulates the transcription of biomarkers involved in the catabolic process (Bonaldo and Sandri, 2013). Phosphorylated FoxO is an inactive form that remains in the cytoplasm without moving into the nucleus. Lack of FoxO translocation into the nucleus prevents the upregulation of atrogin-1, MuRF1, and other target genes involved in the autophagy-lysosomal system which degrades misfolded proteins and organelles, including the mitochondria (Kim and Hwang, 2020; Sa et al., 2017).

Contrarily, proinflammatory cytokines negatively regulate skeletal muscle. Sarcopenia, cachexia, and disuse muscle atrophy are closely associated with inflammation (Caron et al., 2011; Zhou et al., 2016). Proinflammatory cytokines present in the muscle trigger the signaling pathways related to muscle degradation through the NF-κB pathway. This pathway, which upregulates MuRF1 and atrogin-1, facilitates the transcription of proinflammatory cytokines that function as autocrine or paracrine factors (Kim and Hwang, 2020). These proinflammatory cytokines are also involved in muscle cell death through oxidative stress-induced apoptosis (Powers et al., 2016; Sa et al., 2017). Tumor necrosis factor alpha (TNF-α), which inhibits PI3K/Akt/mTOR pathway, is a significant representative of the proinflammatory cytokine (Sa et al., 2017; Wang et al., 2014). Several studies have investigated the anti-inflammatory properties of plants or phytochemicals, considering the role of proinflammatory cytokines in the muscle (Kim and Hwang, 2020; Kim et al., 2020).

### Mitochondrial activity

Mitochondria are double-membrane organelles that have their own genomes (Peterson et al., 2012). Mitochondria play a critical role in adenosine triphosphate (ATP) production through oxidative phosphorylation (OXPHOS), tricarboxylic acid (TCA) cycle, and fatty acid oxidation (Li et al., 2020; Wells et al., 2009). Thus, normal and healthy mitochondria are vital for sustaining muscle function because in terms of muscle contraction, the energy in the form of ATP should be supplied to muscle fibers (Li et al., 2020). Additionally, they not only regulate energy production but also control oxidative stress through the anti-oxidant defense system and are involved in programmed cell death, also called as apoptosis (Ji and Yeo, 2019) Thus, research on muscle atrophy, mainly when caused by aging and muscle disuse, has focused on the mitochondrial function and mitochondrial biogenesis, a process of mitochondria formation (Chang et al., 2019; Kim and Hwang, 2020).

Peroxisome proliferator-activated receptor gamma coactivator 1 alpha (PGC-1α) modulates mitochondrial biogenesis (Ji and Yeo, 2019). Combined with nuclear respiratory factor 1 (NRF-1), PGC-1α increases mitochondrial DNA (mtDNA) by upregulating mitochondrial transcription factor A (Tfam) levels in the nucleus (Zamora and Villena, 2014). Another role of PGC-1α is the inhibition of FoxO, consequently, reducing FoxO-mediated transcription of atrogin-1 and MuRF1 (Ji and Yeo, 2019).

Mitochondria are primary sources of reactive oxygen species (ROS) which are produced during oxidative phosphorylation (OXPHOS) and ATP production. Despite producing ROS, they have their own natural anti-oxidant defense system (Marzetti et al., 2013). However, abnormally accumulated ROS stimulates the release of cytochrome C from mitochondria to the cytosol, thereby initiating mitochondrial-dependent cell death (Powers et al., 2016). Besides apoptosis, the overexpression of ROS decreases the production of ATP by suppressing mitochondrial respiratory chain complexes; therefore, reducing oxidative stress has been suggested as a strategy to prevent muscle atrophy (Huang et al., 2018).

### Myogenesis

Satellite cells, located between the basal lamina and myofiber membrane, are known as muscle stem cells involved in muscle development and muscle regeneration in injured, damaged, and atrophic muscle (Feige et al., 2018; Fukada, 2018). In the formation of myofibers, myogenesis is a necessary process that differentiates satellite cells into myotubes (Fukada, 2018). Myogenesis is a complex process coordinated by myogenic regulatory factors (MRFs), such as Myf5, MyoD, and myogenin (Kim et al., 2019b). Their expression levels, particularly that of MyoD, are governed by upstream elements, including Akt, mTOR, p38, and TAZ (Kim et al., 2017a; Lee et al., 2017a; Zhang et al., 2015). Because myogenesis plays a critical role in muscle formation, regulating myogenesis has been of great interest to counter muscle atrophy (Bonaldo and Sandri, 2013; Fukada, 2018).

## Anti-muscle atrophy properties of flavonoids

**Fig. 2:**
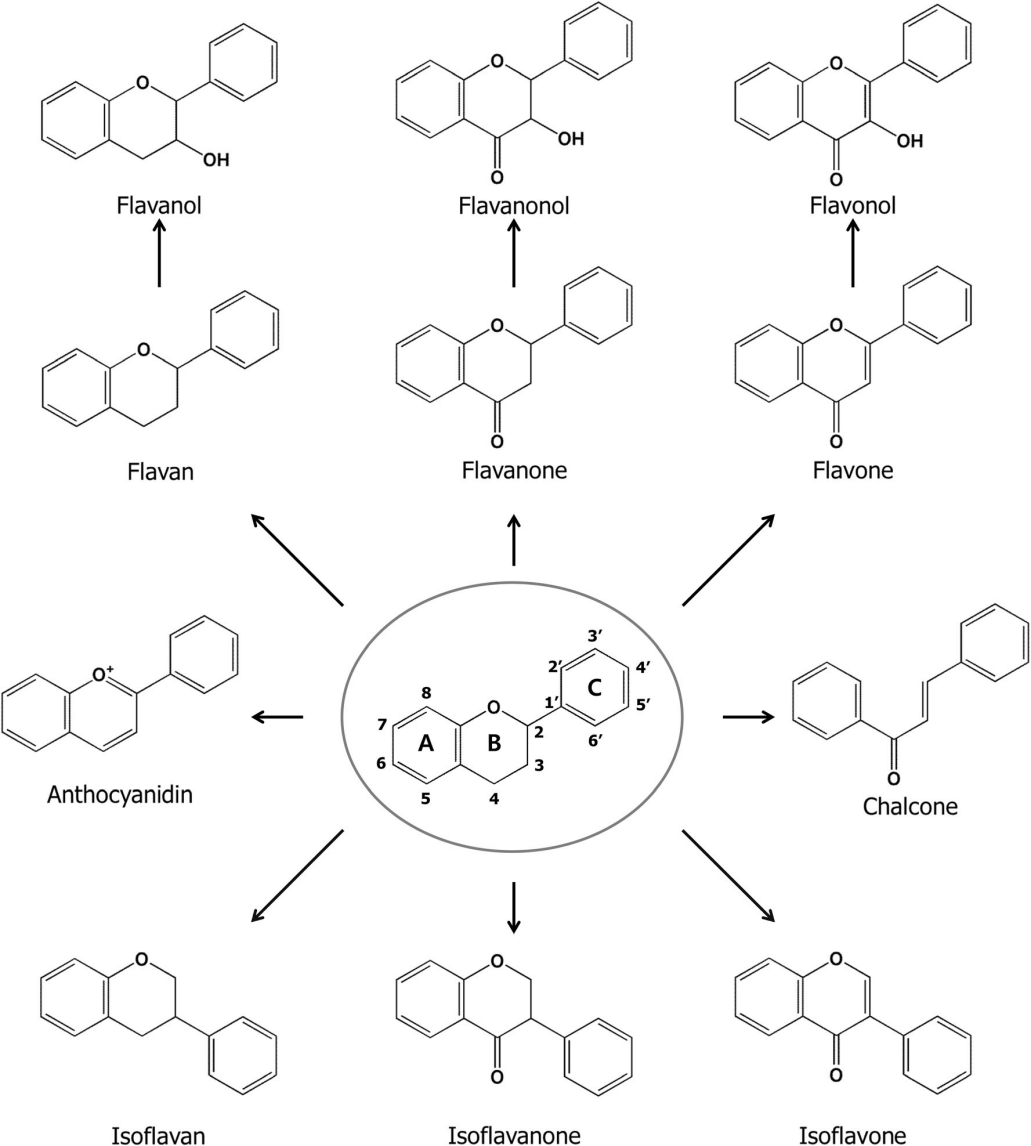
Chemical structures of flavonoid subgroups

Flavonoids are secondary metabolites synthesized by the phenylpropanoid pathway in plants (Kumar and Pandey, 2013; Le Marchand, 2002). The basic structure of flavonoids is a 15-carbon structure (C6–C3–C6) with two benzene rings linked by a 3-carbon chain (Fig. 2) (Kawser Hossain et al., 2016). The subclasses of flavonoids are based on oxidation, hydroxylation, and substitution characteristics and include flavan, flavanol, flavanone, flavanonol, flavone, flavonol, isoflavan, isoflavanone, isoflavone, anthocyanidin, and chalcone (Fig. 2). Although flavonoids exist as aglycones, there are glycosylated, methylated, and prenylated flavonoids in nature (Kumar and Pandey, 2013). The biological activities exhibited by flavonoids are as diverse as their structures (Kumar and Pandey, 2013). Flavonoids exert positive effects on several organs, such as brain (Spencer, 2008), bone (Welch and Hardcastle, 2014), skin (Saraf et al., 2007), and liver (Kumar and Pandey, 2013). In addition, they are beneficial against cancer (Le Marchand, 2002), metabolic diseases (Kawser Hossain et al., 2016), cardiovascular disease (van Dam et al., 2013), osteoporosis (Welch and Hardcastle, 2014), and urolithiasis (Zeng et al., 2019). Moreover, several biological activities of flavonoids against bacteria (Farhadi et al., 2019), inflammation (Pan et al., 2010; Serafini et al., 2010), and oxidative stress (Procházková et al., 2011) have been well explored. Specifically, multiple in vitro and in vivo studies have suggested that flavonoids are potential therapeutics for treating muscle atrophy, and these studies have elucidated some of their underlying mechanisms. Table 1 summarizes the effects of several flavonoid compounds on muscle atrophy along with their underlying molecular mechanisms.

**Table 1:** Flavonoid compounds having potential anti-atrophic effect on muscle

Subclass	Chemical name	Source	Model	Inducer of muscle atrophy	Mechanism	References
Flavanol		*Theobroma cacao* L., *Camellia sinensis* L.	C2C12 myotubes	–	MHC↑, MyoD↑, Myogenin↑, Akt↑	Lee et al. (2017a)
				Clinorotation	MuRF1↓, Atrogin-1↓	Hemdan et al. (2009)
			C57/BL6 mice	Aged (1-year old)	Plantar muscle area↑, Tfam↑, Running time↑, Mitochondria density↑	Nogueira et al. (2011)
				Aged (20-month old)	Survival rate↑, Muscle degradation↓	Si et al. (2019)
				Aged (26-month old)	SOD↑, Catalase↑, NRF2↑, Tfam↑, PGC-1α↑	Moreno-Ulloa et al. (2015)
				Aged (26-month old)	Myf5↑, MyoD↑, Myogenin↑, Myostatin↓	Gutierrez-Salmean et al. (2014)
			ICR mice	Aged (24-month old)	MyoD↑, SOD↑, MuRF1↓, mTOR↑	Hong et al. (2020)
			Elder people	Aged (62-years old)	Grip strength↑	Gutierrez-Salmean et al. (2014)
Flavanol		*Camellia sinensis* L.	C2C12 myotubes	–	MHC↑, Myogenin↑, MyoD↑, Myotube diameter↑	Kim et al. (2017b)
				Clinorotation	MuRF1↓, Atrogin-1↓	Hemdan et al. (2009)
			Satellite cells	–	Myogenesis↑, Myf5↑, Akt↑	Kim et al. (2017b)
			C57/BL6 mice	Cardiotoxin	Myf5↑, MyoD↑, Pax7↑	Kim et al. (2017b)
Flavanol		*Camellia sinensis* L.	C2C12 myotubes	–	Myotube formation↑, MHC↑, Myogenin↑, Myf5↑	Kim et al. (2017b)
				Serum-free starvation	Atrogin-1↓, MuRF1↓, Protein synthesis↑	Mirza et al. (2014)
				Clinorotation	Atrogin-1↓, MuRF1↓	Hemdan et al. (2009)
				TNF-a	Protein synthesis↑, Protein degradation↓	Mirza et al. (2014)
			SAMP8 mice	Aging	IGF-1↑, NRF1↑, Tfam↑, mtDNA↑	Liu et al. (2015)
			C57BL/6 mice	Cardiotoxin	Fiber size↑, Myf5↑	Kim et al. (2017b)
				Lewis lung cell-induced tumor	Muscle mass↑, MuRF1↓, Atrogin-1↓	Wang et al. (2011)
			Wistar rats	Sciatic nerve injury	Muscle function↑, Muscle fiber repair↑	Renno et al. (2012)
			FBN rats	Aged (34-month old) + Hindlimb suspension	Plantar muscle mass↑, force↑, PGC-1α↑, SOD↑, Akt↑, Bax↓, Bcl2↑, Caspase 3↓, Beclin1↓, LC3↓	Alway et al. (2014); Takahashi et al. (2017)
			SD rats	Aged (20-month old)	Muscle mass↑, IGF-1↑, MuRF1↓, Atrogin-1↓	Meador et al. (2015)
Flavanone		*Hunulus lupulus* L.	C2C12 myotubes	–	Akt↑, PI3K↑, p70S6K↑	Mukai et al. (2016a)
			C57BL/6 mice	Scatic nerve enervation	Gastrocnemius muscle mass↑, Akt↑, Atrogin-1↓	Mukai et al. (2012)
				Cast immobilization	Tibialis anterior muscle mass↑, Akt↑, IGF↑	Mukai et al. (2016a)
				Ovariectomy	Tibialis anterior muscle mass↑	Mukai et al. (2016a)
Flavanonol		*Ampelopsis grossedentata*	L6 myotubes	Dexamethasone	Myotube diameter↑, Mitochondrial contents↑, Tfam↑, PGC-1α↑, Akt↑, mTOR↑, Atrogin-1↓	Huang et al. (2018)
			SD rats	Dexamethasone	Gastrocnemius muscle mass↑, Grip strength↑, Cross-sectional area↑, PGC-1α↑, Tfam↑, ATP↑, MuRF1↓, Atrogin-1↓, Akt↑, mTOR↑	Huang et al. (2018)
				D-galactose	Gastrocnemius mass↑, Cross-sectional area↑, PGC-1α↑, Beclin1↑, LC3↑, Atrogin-1↓, Myostatin↓	Kou et al. (2017)
Flavanonol		*Silybum marianum*	C2C12 myotubes	Cancer cell-cultured medium	Protein contents↑, MuRF1↓, Atrogin-1↓	Shukla et al. (2015)
			NCr-nu/nu mice	S2-013 cancer cell-induced tumor	Gastrocnemius muscle mass↑, Grip strength↑, MuRF1↓, Atrogin-1↓	Shukla et al. (2015)
Flavone		*Kaempferia Parviflora*	C57BL/6J mice	Aged (18-month old)	Gastrocnemius mass↑, Cross-sectional area↑, MuRF1↓, Atrogin-1↓, Akt↑, mTOR↑, PGC-1α↑	Kim and Hwang (2020)
Flavone		Vegetable	C2C12 myotubes	–	Myogenesis↑, MHC↑, IGF-1↑, MyoD↑, Akt↑, p70S6K↑	Jang et al. (2017)
				LPS	Myotube diameter↑, Atrogin-1↓	Shiota et al. (2015)
				Palmitic acid	Myotube diameter↑, MuRF1↓, Atrogin-1↓, MHC↑, OXPHOS↑	Choi et al. (2017)
			C57BL/6 mice	–	Quadriceps muscle mass↑, Running distance↑, Cross-sectional area↑, MHC↑, PGC-1α↑	Jang et al. (2017)
				Sciatic nerve denervation	Gastrocnemius muscle mass↑, MHC↑, MuRF1↓, Atrogin-1↓, TNF-α↓, IL-6↓	Choi et al. (2018)
				High-fat diet	Cross-sectional area↑, mtDNA↑, Quadriceps muscle mass↑, MuRF1↓, Atrogin-1↓, TNF-α↓, IL-6↓, IL-1β↓, PGC-1α↑, Tfam↑	Choi et al. (2017)
Flavone		*Scutellaria baicalensis*	BALB/c mice	CT26 colon cancer cell-induced tumor	Gastrocnemius muscle mass↑, Fiber size↑, TNF-α↓, IL-6↓, Atrogin-1↓, MuRF1↓	Li et al. (2014)
			Human	Head and neck cancer	Lean body mass↑, NF-κB↓, Activin A↓	Emanuele et al. (2016)
Flavone		Vegetable	C2C12 myotubes	LPS	Myotube diameter↑, Atrogin-1↓	Shiota et al. (2015)
			Wistar rats	Dexamethasone	Gastrocnemius muscle mass↑, Muscle strength↑, GSH↑, Caspases-3↑	Hawila et al. (2019)
			C57BL/6 mice	Lewis lung cancer cell-induced tumor	Mscle mass↑, Atrogin-1, IL-6↓, MuRF1↓, TNF-α↓	Chen et al. (2018b)
Flavone		*Citrus sienesis*	Satellite cells from SD rats	Aging	Myoblast length↑, MyoD↑, Myogenin↑	Kim et al. (2019c)
Flavonol		*Epimedium koreanum*	C2C12 myotubes	–	PI3K↑, Akt↑, mTOR↑, p70S6K↑, 4EBP1↑, MuRF1↓, Atrogin-1↓	Zhang et al. (2016)
			SD rats	Tail-suspension	Soleus muscle mass↑, Cross-sectional area↑, mTOR↑, p70S6K↑, 4EBP1↑, MuRF1↓, Atrogin-1↓	Zhang et al. (2016)
Flavonol		*Prunus dulcis*	C57BL/6J mice	Lewis lung carcinoma cell induced tumor	Cross-sectional area↑	Yoshimura et al. (2018)
Flavonol		*Allium cepa*	C2C12 myotubes	Palmitic acid and macrophage cultured medium	TNF-α↓, MuRF1↓, Atrogin-1↓	Le et al. (2014)
				TNF-α	Myotube diameter↑, MuRF1↓, Atrogin-1↓	Kim et al. (2018b)
				Clinorotation	MuRF1↓, Atrogin-1↓	Hemdan et al. (2009)
				Dexamethasone	Akt↑, MuRF1↓, Atrogin-1↓	Otsuka et al. (2019)
			C57BL/6J mice	High-fat diet	Gastrocnemius muscle mass↑, Atrogin-1↓, Muscle fiber diameter↑, TNF-α↓, MuRF1↓	Le et al. (2014)
					MuRF1↓, NRF2↑, NF-κB↓	Kim et al. (2018b)
				Tail-suspension	Gastrocnemius muscle mass↑ MuRF1↓, Atrogin-1↓	Mukai et al. (2010)
				Sciatic nerve denervation	Gastrocnemius muscle mass↑, Cross-sectional area↑, Akt↑, IGF-1↑	Mukai et al. (2016b)
			Apc^Min/+^ mice	Adenomatous polyposis coli gene mutation	Gastrocnemius muscle mass↑, Quadriceps muscle mass↑, Grip strength↑, IL-6↓	Velázquez et al. (2014)
			Nude mice	A549 lung cancer cell-induced tumor	Gastrocnemius muscle mass↑, MuRF1↓, MHC↑, DNA damage↓, TNF-α↓, IL-6↓, Atrogin-1↓	Chan et al. (2018)
			BALB/cCrS1c mice	Dexamethasone	Gastrocnemius muscle mass↑, MuRF1↓, Atrogin-1↓, Myostatin↓, Akt↑	Otsuka et al. (2019)
Isoflavan		*Glycyrrhiza glabra* L.	C2C12 myotubes	Dexamethasone	Protein degradation↓, MuRF1↓, Cbl-b↓, FoxO3↓	Yoshioka et al. (2019)
			C57BL/6J mice	Dexamethasone	Tibialis anterior muscle mass↑, Gastrocnemius muscle mass↑, MuRF1↓, Cbl-b↓, FoxO3↓, GR↓	Yoshioka et al. (2019)
Isoflavone		*Psoralea coryliforlia*	C2C12 myotbue	Dexamethasone	Myotube diameter↑, MyoD↑, MHC↑, Myogenin↑, NF-κB↓, MuRF1↓, Atrogin-1↓	Han et al. (2020)
Isoflavone		*Glycine max*	C2C12 myotubes	TNF-α	MuRF1↓, SIRT1↑	Hirasaka et al. (2013)
				Dexamethasone	Myotube diameter↑, Akt↑, MHC↑, Myogenin↑, MyoD↑	Lee et al. (2017b)
				LPS	Atrogin-1	Shiota et al. (2015)
				–	Myotube number↑, mTOR↑, Akt↑, p70S6K↑, MHC↑, MyoD↑, Myogenin↑	Lee et al. (2017b), Zheng et al. (2018)
Isoflavone		*Glycine max*	C2C12 myotubes	TNF-α	Myotube diameter↑, MuRF1↓	Hirasaka et al. (2013)
				LPS	Atrogin-1↓	Shiota et al. (2015)
				–	Myotube diameter↑, IGF↑, MHC↑	Zheng et al. (2018)
			L6 myotube	–	Protein synthesis↑	Jones et al. (2005)
			Wistar rats	Sciatic nerve denervation	Soleus muscle mass↑, FoxO1↑	Aoyama et al. (2016)
Antho-cyanidin		*Vaccinium* Spp.	C2C12 myotubes	Dexamethasone	Cbl-b↓, MuRF1↓, NFATc3↑, miR-23a↑	Murata et al. (2016; 2017)
			C57BL/6J mice	Tail-suspension	Quadriceps muscle mass↑, Cbl-b↓, miR-23a↑, Gastrocnemius muscle mass↑, MuRF1↓	Murata et al. (2016; 2017)
Chalcone		*Angelica keiskei*	C2C12 myotubes	–	Myogenesis↑, MyoD↑, Myogenin↑, MHC↑	Kweon et al. (2019)
				Dexamethasone, cancer cell cultured media	MuRF1↓, MHC↑, Atrogin-1↓, Myostatin↓	Kweon et al. (2019)
Chalcone		*Psoralea corylifolia*	C2C12 myotubes	–	MyoD↑, MHC↑	Han et al. (2020)
				TNF-α	Myotube diameter↑, MyoD↑, MHC↑, Myogenin↑, NF-κB↓, NRF2↑, MuRF1↓, Atrogin-1↓	Hur et al. (2019)
Chalcone		*Boesenbergia pandurata*	L6 myotube	TNF-α	Myotube diameter↑, MyoD, Myogenin↑, PI3K↑, Akt↑, mTOR↑, Atrogin-1↓, MuRF1↓, Beclin1↓, LC3↓, ROS↓, Catalase↑, SOD↑	Sa et al. (2017)

### Flavanol

#### Epicatechin

Many types of catechins have been identified in cocoa (*Theobroma cacao* L.) and green tea (*Camellia sinensis* L.) with epicatechin as the smallest molecule (Li et al., 2020). In vitro studies with epicatechin have focused on elucidating the molecular mechanisms of myogenesis (Kim et al., 2017b), muscle growth (Moreno-Ulloa et al., 2018), and mitochondrial functions (Chang et al., 2017). One paper reported the anti-atrophic effect of epicatechin on skeletal muscle using C2C12 myotubes subjected to clinorotation; this effect was demonstrated as the downregulation of atrogin-1 and MuRF1 by dephosphorylating the extracellular signal-regulated kinase (ERK) (Hemdan et al., 2009). However, in dexamethasone- and lipopolysaccharide (LPS)-treated C2C12 myotubes, epicatechin has exhibited no effect on the expression of MuRF1 or atrogin-1 (Hemdan et al., 2009; Shiota et al., 2015). On myogenesis, epicatechin promoted muscle differentiation by upregulating the protein expression of myogenic markers, such as MyoD, myogenin, and MHC in C2C12 muscle cells, MyoD-transfected 10T/C embryonic fibroblasts, and human rhabdomyosarcoma cells (Lee et al., 2017a). Akt and p38 have been involved at the early stage of the epicatechin-mediated muscle differentiation; however, epicatechin primarily activated Akt at a late stage. Epicatechin also improved mitochondrial functions, as evidenced by increases in mitochondria-related gene expression, enzyme activities, and ATP levels in response to epicatechin treatment (Chang et al., 2017). Similarly, Moreno-Ulloa et al. (2018) reported that epicatechin stimulated mitochondrial biogenesis through the G-protein-coupled estrogen receptor, Tfam, nuclear respiratory factor (NRF)-2, and citrate synthase. The results of in vitro experiments showed the beneficial effects of epicatechin on muscle wasting and muscle function. Additionally, epicatechin increased the mRNA expression of MyoD, superoxide dismutase (SOD), and catalase in the biceps femoris muscle and decreased the expression of FoxO3, myostatin, and MuRF1 in the soleus muscle (Hong et al., 2020). Si et al. (2019) observed that epicatechin increased the survival rate of aged mice and delayed the degeneration of skeletal muscle. Furthermore, epicatechin gave rise to a positive effect on the skeletal muscle in 26-month-old C57BL/6 mice (Moreno-Ulloa et al., 2015). Apart from its anti-oxidant activity, as demonstrated by increases in glutathione (GSH)/glutathione disulfide (GSSG) levels and protein expression of anti-oxidant enzymes, SOD, catalase, and glutathione peroxidase (GPx), epicatechin also stimulated mitochondrial biogenesis by upregulating sirtuin 1 (SIRT1), PGC-1α, NRF-2, Tfam, and genes related to mitochondrial function in aged skeletal muscle. Consistently, epicatechin increased both exercise time and distance as well as the area and perimeter of the plantaris muscle by upregulating Tfam and increasing mitochondrial volume in 1-year-old C57BL/6 mice (Nogueira et al., 2011). Finally, epicatechin increased Myf5 and MyoD, stimulated myogenesis, and decreased myostatin in skeletal muscle of aged mice (Gutierrez-Salmean et al., 2014). In clinical trials with the elderly, treatment with epicatechin for 7 days increased the hand grip strength along with the reduced levels of myostatin and follistatin.

#### Epicatechin gallate

Epicatechin gallate (ECG) is a catechin compound found in green tea (*C. sinensis* L.) (Li et al., 2020). It stimulates myogenesis, as evidenced by an increase in Myf5, MyoD, and myogenin expression through Akt activation in response to ECG treatment (Kim et al., 2017b). Another study reported that ECG increased myotube length, diameter, and number by upregulating myoD, myogenin, and MHC (Li et al., 2019). In cardiotoxin-damaged muscle, ECG increased muscle fiber size by increasing myf5 but did not affect MyoD and Pax7 (Kim et al., 2017b). Additionally, ECG downregulated MuRF1 and atrogin-1 expression in C2C12 myotubes subjected to clinorotation but did not affect the dexamethasone-treated C2C12 myotubes (Hemdan et al., 2009).

#### Epigallocatechin-3-gallate

Epigallocatechin-3-gallate (EGCG) is a primary polyphenol found in green tea (*C. sinensis* L.) (Mirza et al., 2014). The effect of EGCG on muscle atrophy has been investigated in multiple in vitro models using various methods to induce muscle atrophy, including starvation, TNF-α, dexamethasone, and clinorotation treatments. Although EGCG did not affect dexamethasone-treated C2C12 myotubes (Hemdan et al., 2009), it increased protein synthesis and decreased protein degradation in TNF-α-treated C2C12 myotubes (Mirza et al., 2014). EGCG increased protein synthesis and degradation in C2C12 myotubes subjected to starvation by downregulating MuRF1 and atrogin-1 (Mirza et al., 2014). It consistently reduced atrogin-1 and MuRF1 mRNA expression by decreasing the phosphorylation of ERK protein in clinorotation-treated C2C12 myotubes (Hemdan et al., 2009). Besides inhibiting muscle atrophy, EGCG also stimulated myogenic differentiation by increasing Myf5, MyoD and myogenin expression in in vitro models; however, it did not upregulate Pax7 (Kim et al., 2017a; 2017b). EGCG also enhanced mitochondrial function, as evidenced by increases in ATP levels and mitochondrial protein levels (Chang et al., 2017).

The anti-atrophic effect of EGCG on muscle has been studied in various animal models of sarcopenia, cachexia, and disuse muscle atrophy. Wang et al. (2011) examined the anti-cachectic effect of EGCG in Lewis lung cancer (LLC) tumor-bearing mice. In this model, pretreatment with EGCG increased body weight and the mass of the gastrocnemius muscle by reducing MuRF1, atrogin-1, and NF-κB protein expression. In another study, after a sciatic nerve crush injury, the oral administration of EGCG improved skeletal muscle function, repaired the damaged muscle fibers, and had a significant anti-apoptotic effect (Renno et al., 2012). EGCG has been frequently studied for its impact on sarcopenia. EGCG supplementation increased the gastrocnemius muscle mass and the cross-sectional area of muscle fibers in 20-month-old Sprague–Dawley (SD) rats by downregulating MuRF1, atrogin-1, and myostatin and increasing IGF-1 mRNA expression (Meador et al., 2015). In senescence-accelerated mice, EGCG increased p-Akt, NRF-1, and Tfam expression and restored mtDNA copy number but did not affect PGC-1α in the skeletal muscle (Liu et al., 2015). Among the studies on sarcopenia, some have focused on the disuse-treated sarcopenic model. EGCG did not prevent unloading-induced muscle atrophy in aged mice (Alway et al., 2014). However, it stimulated the recovery of the muscle, as demonstrated by increases in the weight and cross-sectional area of the plantaris muscle tissue. In this model, EGCG activated Akt, increased the number of satellite cells, and decreased apoptosis-related markers, such as caspase-3 and Bax. Moreover, EGCG not only suppressed the autophagy-related proteins, Beclin1 and microtubule-associated protein light chain 3 (LC3) but also induced PGC-1α in the reloaded muscles of aged rats (Takahashi et al., 2017).

### Flavanone

#### 8-Prenylnaringenin

8-Prenylnaringenin found in hop (*Humulus lupulus*) is a prenylated flavanone where the hydrogen atom at the 8-position of naringenin is substituted with a prenyl group (Mukai et al., 2012; 2016a). In C2C12 myotubes, 8-prenylnaringenin increased the levels of p-Akt, p-PI3K, and p-p70S6K (Mukai et al., 2016a). Mukai et al. (2012) investigated whether 8-prenylnaringenin or naringenin prevented muscle atrophy in mice. A diet containing 8-prenylnaringenin increased the mass of the gastrocnemius muscle by stimulating the phosphorylation of Akt and downregulating atrogin-1. However, a diet containing naringenin had no effect on muscle atrophy. When flavonoid levels in the gastrocnemius muscle tissue were measured, the level of 8-prenylnaringenin was ten times higher than that of naringenin, suggesting that prenylation increases the accumulation of 8-prenylnaringenin in muscle tissue and improves skeletal muscle physiology. These results indicate that the prenyl group is a critical component of the anti-atrophic effect of 8-prenylnaringenin on the muscle. Likewise, 8-prenylnaringenin not only improved the recovery activity of muscle mass, as demonstrated by an increase in the weight of the tibialis anterior muscle after cast immobilization, but also increased muscle mass in ovariectomized mice (Mukai et al., 2016a). Unlike the positive effect of 8-prenylnaringenin on muscle, naringenin delayed muscle differentiation by inactivating estrogen receptor (ER) α-mediated Akt phosphorylation (Pellegrini et al., 2014). Previous studies have reported the phytoestrogenic activity of 8-prenylnaringenin (Coldham and Sauer, 2001; Milligan et al., 2000), suggesting that ER-mediated cellular signaling pathways are involved in the improvement of muscle atrophy in response to 8-prenylnaringein (Mukai et al., 2012). However, the molecular mechanisms underlying these effects of 8-prenylnaringenin are still unclear.

### Flavanonol

#### Dihydromyricetin

Dihydromyricetin, also called ampelopsin, is a flavanonol found in rattan tea (*Ampelopsis grossedentata*), an edible plant in China (Huang et al., 2018; Kou et al., 2017). Huang et al. (2018) investigated the anti-atrophic effect of dihydromyricetin using a dexamethasone-induced muscle atrophy model. An in vivo study showed that the oral administration of dihydromyricetin for 2 weeks increased muscle weight, the cross-sectional area of muscle fibers, grip strength, which is a biomarker representing muscle function, and mitochondria content as well as improved mitochondrial morphology through the PGC-1α/Tfam and PGC-1α/mitofusin 2 (Mfn2) signaling pathways. These results were consistent with those of an in vitro study wherein dihydromyricetin increased myotube diameter, mitochondrial mass, and relative mtDNA content by stimulating PGC-1α-mediated signaling pathways in dexamethasone-treated L6 myotubes. Dihydromyricetin also increased the Akt/mTOR pathway for protein synthesis and decreased FoxO3-regulated proteolysis.

D-Galactose (Gal) treatment stimulated the aging process by increasing oxidative stress in rodents, thus resulting in deficient autophagy system and excessive apoptosis (Fan et al., 2017). Dihydroxymyricetin attenuated muscle atrophy in D-gal-treated rats (Kou et al., 2017). Specifically, dihydromyricetin treatments at 100 and 200 mg/kg for 6 weeks significantly increased the ratio of the gastrocnemius muscle to body weight by stimulating the AMP-activated protein kinase (AMPK)/SIRT1/PGC-1α pathway, activating autophagy and inhibiting apoptosis and ubiquitin–proteasome system. These results suggest that dihydromyricetin attenuates muscle atrophy through the PGC-1α signaling pathway.

#### Silibinin

Silibinin, also known as silybin, is a compound found in milk thistle (*Silybum marianum*) (Bosch-Barrera and Menendez, 2015). Structurally, silibinin is a flavononol compound conjugated with lignan; therefore, it is also called flavonolignan (Kauntz et al., 2012). Although it is well-known for its anti-cancer property (Bosch-Barrera and Menendez, 2015; Kauntz et al., 2012), only one study examined the inhibitory effect of silibinin on cancer-induced cachexia (Shukla et al., 2015). Shukla et al. (2015) reported that when C2C12 myotubes were cultured in S2-013 pancreatic cell-conditioned medium, MuRF1 and atrogin-1 expression was upregulated and protein content was decreased; however, silibinin treatment recovered protein content and decreased MuRF1 and atrogin-1 expression. In S2-013 tumor-bearing mice, silibinin treatment reduced the tumor weight, volume, and gastrocnemius muscle wasting and increased the grip strength and latency to fall. In the gastrocnemius muscle tissue, MuRF1, atrogin-1, interleukin (IL)-6, and TNF-α expression was significantly reduced by silibinin treatment. Thus, this anti-cachectic effect of silibinin resulted from the downregulation of MuRF1, atrogin-1, IL-6, and TNF-α expression in the gastrocnemius muscle tissue.

### Flavone

#### 5,7-Dimethoxyflavone

5,7-Dimethoxyflavone is found in the rhizome of black ginger (*Kaempferia parviflora*) (Lee et al., 2018). The anti-sarcopenic effect of 5,7-dimethoxyflavone was studied in 18-month-old C57BL/6J mice. Oral administration of 5,7-dimethoxyflavone at 25 and 50 mg/kg/day for 8 weeks improved the exercise capacity and grip strength and increased muscle weight and volume (Kim and Hwang, 2020). At the molecular level, 5,7-dimethoxyflavone increased the mTOR pathway and decreased MuRF1 and atrogin-1-mediated proteolysis through the PI3K/Akt pathway in the gastrocnemius muscle. Moreover, 5,7-dimethoxyflavone increased mtDNA content through PGC-1α. Moreover, 5,7-dimethoxyflavone enhanced energy metabolism by upregulating PGC-1α and increased the glycogen content and the mRNA expression of glycogen synthase in C2C12 myocytes (Toda et al., 2016a; 2016b). Although the anti-atrophic effects of 5,7-dimethoxyflavone on skeletal muscle in cachexia and obesity have not been reported, some studies have demonstrated that it showed anti-obesity (Song et al., 2016) and anti-cancer (Li et al., 2017; Yang et al., 2012) activities, thus implying its therapeutic effect on several types of muscle atrophy.

#### Apigenin

Apigenin is a 5,7,4′-trihydroxyflavone found in edible plants, such as parsley, celery, and grapefruit (Jang et al., 2017). Apigenin treatment inhibited LPS-induced atrogin-1 expression in C2C12 myotubes by reducing the phosphorylation of c-Jun N-terminal protein kinase (JNK), thereby increasing the myotube diameter; however, 5,7-dihydroxychromone showed no effect. Interestingly, 5,7-dihydroxychormone has the similar structure as apigenin, except that apigenin has a phenyl group at the 2-position (Shiota et al., 2015). These results indicate that the reduction in atrogin-1 expression in response to apigenin treatment is due to its phenyl group. In C2C12 cells, apigenin also increased the palmitic acid-reduced myotube diameter by downregulating MuRF1 and improving mitochondrial function through oxidative phosphorylation (OXPHOS)-involved markers (Choi et al., 2017). In the animal model for obesity-induced muscle atrophy, a high-fat diet containing apigenin increased muscle mass, the cross-sectional area of muscle fibers, and running distance by downregulating MuRF1 and atrogin-1 expression; furthermore, it reduced the levels of TNF-α and IL-6 in the serum and the gastrocnemius muscle tissue (Choi et al., 2017). Besides, apigenin reduced mitochondrial dysfunction by stimulating citrate synthases, complex I, and complex II activities and upregulating succinate dehydrogenase complex subunits (SDH) B, SDHD, and ubiquinol-cytochrome C reductase core protein 1 (UQCRC1). Apigenin also stimulated mitochondrial biogenesis through PGC-1α and Tfam mRNA expression. The denervation of the sciatic nerve decreased the area of the muscle fibers; however, a diet containing apigenin increased the muscle fiber area and the weight of the gastrocnemius and soleus muscles (Choi et al., 2018). At a molecular level, apigenin upregulated MHC, downregulated MuRF1, and decreased TNF-α expression in the gastrocnemius muscle tissue. In the soleus muscle, apigenin increased MHCIIa expression and reduced TNF-α and IL-6 expression. Apart from inhibiting muscle atrophy, apigenin induced muscle hypertrophy and myogenic differentiation by stimulating the Akt/p70S6K/4EBP-1 pathway and myoD protein expression, respectively (Jang et al., 2017). Apigenin treatment notably increased the thickness of C2C12 myotubes and running distance and the weight of quadriceps muscle in C57BL/6 mice. These results suggest that apigenin has the therapeutic potential for inhibiting muscle atrophy and inducing muscle hypertrophy.

#### Baicalin

Baicalin is a glycosidic flavone derived from *Scutellaria baicalensis* and has anti-inflammatory and anti-cancer properties (Emanuele et al., 2016). In CT26 adenocarcinoma-bearing mice or a preclinical model for cancer cachexia, an intraperitoneal injection of 15 or 50 mg/kg baicalin increased the weight and cross-sectional area of the gastrocnemius muscle and alleviated anorexia, an inability to eat (Li et al., 2014). In this model, baicalin treatment significantly decreased MuRF1 and atrogin-1 protein expression by inactivating the NF-κB pathway in the gastrocnemius muscle tissue and reducing the serum levels of TNF-α and IL-6. A clinical trial with head and neck cancer patients showed that a daily intake of 50 mg of baicalin for 3 months increased the lean body mass and decreased the serum levels of NF-κB and activin A which are factors negatively affecting the muscle mass (Emanuele et al., 2016). These results indicate that the inactivation of NF-κB mediates the inhibitory effect of baicalin on muscle wasting.

#### Luteolin

Luteolin is a 3′,4′,5,7-tetrahydroxyflavone found in edible plants, such as fruits and vegetables (Hawila et al., 2019). Three researches (one in vitro and two in vivo studies) have suggested that luteolin is a protective agent against muscle atrophy. In LPS-stimulated C2C12 myotubes, luteolin significantly increased the myotube diameter by downregulating atrogin-1 (Shiota et al., 2015). 5,7-Dihydroxychromone has a chemical structure wherein the phenyl ring at the 2-position of luteolin is substituted by hydrogen. This compound did not affect LPS-induced atrogin-1 expression. This finding indicates that the phenyl ring in luteolin is essential for regulating atrogin-1 in LPS-treated C2C12 myotubes. Two studies have investigated the molecular mechanisms of luteolin-induced inhibition of muscle atrophy in different animal models: a LLC tumor-bearing mouse model for cachexia (Chen et al., 2018b) and a dexamethasone-treated mouse (Hawila et al., 2019). In the cachexia model, luteolin increased the weight of the gastrocnemius muscle, tibialis anterior muscle, and heart (Chen et al., 2018b). It was reported that a decrease in MuRF1 and atrogin-1 expression was due to a reduction in NF-κB and p38 expression, respectively, in the muscle of luteolin-treated mice. The serum levels of IL-6 and TNF-α were also reduced by luteolin. Luteolin treatment increased grip strength, the cross-sectional area of muscle fibers, and the mass of the gastrocnemius muscle in dexamethasone-induced muscle atrophy model (Hawila et al., 2019). The results of this study suggest that its anti-apoptotic and anti-oxidant mechanisms are involved in the inhibition of muscle atrophy in response to luteolin treatment, as evidenced by decreased caspase-3 and increased GSH, respectively. Consequently, luteolin hinders the process of muscle atrophy by inhibiting the ubiquitin–proteasome system, apoptosis, inflammatory responses, and oxidative stress.

#### Sinensetin

Sinensetin, found in cat’s whiskers (*Orthosiphon stamineus*) and Citrus species including *Citrus sinensis*, is 3′,4′,5,6,7-pentamethoxyflavone (Akowuah et al., 2005; Ooghe et al., 1994). Sinensetin has anti-inflammatory activity, as demonstrated by its inhibition of cyclooxygenase-2 (COX-2), inducible nitric oxide synthase (iNOS), and NF-κB in LPS-treated L6 myotubes (Kim et al., 2019a). The ability of sinensetin to suppress sarcopenia was examined in satellite cells isolated from the thigh and calf muscles of rats (Kim et al., 2019b). In this ex vivo study, the differentiation of satellite cells isolated from the muscle tissue was lesser in old rats than in young rats. However, sinensetin treatment recovered the differentiation ability of these cells in old rats to a level similar to that in young rats. This recovery was associated with an increase in the protein expression of MyoD and myogenin.

### Flavonol

#### Icaritin

Icariin is a prenylated flavonol glycoside found in *Epimedium koreanum*. Icaritin is usually found in the intestine after *E. koreanum* or icariin is orally administered to animals, indicating that icaritin is a metabolite of icariin (Liu and Lou, 2004). Although there is no research on the effect of icariin on muscle atrophy, one study investigated the inhibitory effect of icaritin on unloading-induced muscle atrophy in rats (Zhang et al., 2016). In this study, the activity of icaritin on C2C12 myotubes was first presented before evaluating the inhibitory effect of icaritin on muscle atrophy in rats. The in vitro test showed that icaritin stimulated the mTOR/p70S6K/4EBP-1 pathway and reduced the relocation of FoxO from the cytosol to the nucleus. In an animal study, SD rats were subjected to tail-suspension to induce muscle atrophy. A high concentration of icaritin treatment (120 mg/kg/day) significantly increased the ratio of the soleus muscle to body weight and the cross-sectional area of myofibers and improved force and concentration-relaxation time relative to untreated control animals. At the molecular level, icaritin stimulated the mTOR/P70S6K/4EBP-1 pathway and reduced the nuclear translocation of FoxO1 and FoxO3, which are consistent with the results of the in vitro experiment. Icaritin also activated the PI3K/Akt pathway in C2C12 cells and the soleus muscle tissue. Co-treatment with icaritin and wortmannin, a specific inhibitor of the p110 catalytic subunit of PI3K, abolished all effects of icaritin on muscle atrophy. Taken together, in terms of the attenuating effect of icaritin on muscle atrophy, icaritin targets the PI3K/Akt pathway.

#### Morin

Morin is a flavonol compound found in multiple plants, such as almond hulls, seaweeds, and guava (Yoshimura et al., 2018). In LLC tumor-bearing mice, a diet containing 0.1% morin increased the weight and cross-sectional area of muscle. However, morin treatment did not affect protein synthesis in C2C12 cells. It was suggested that the anti-cachectic effect of morin is due to its anti-proliferative activity in LLC cells. However, it is still unknown how morin intake regulates the molecular mechanisms of anti-cachectic activity in muscle tissue.

#### Quercetin

Quercetin is mainly found in onions (Mukai et al., 2016b). A review of the anti-atrophic effects of quercetin on muscle from the perspective of its anti-oxidant activities was reported (Mukai and Terao, 2013). The ability of quercetin to inhibit muscle atrophy or wasting has been studied in numerous models for obesity, muscle disuse, and cachexia.

Quercetin has exhibited an anti-obesity effect in different models (Rivera et al., 2008; Seo et al., 2015). Due to this effect, some studies have examined the effect of quercetin on muscle atrophy in obese mice. Quercetin reduced epididymal fat and increased the weight of the quadriceps and gastrocnemius muscle tissues in high-fat diet-induced obese mice, suggesting that quercetin has an anti-obesity effect and reduces muscle atrophy (Le et al., 2014). Quercetin decreased atrogin-1 and MuRF1 expression in the gastrocnemius muscle of obese mice and reduced TNF-α and monocyte chemoattractant protein-1 (MCP-1) transcripts. It also reduced atrogin-1 and MuRF1 mRNA levels in C2C12 myotubes cocultured with RAW264.7 macrophages along with palmitic acid treatment. Kim et al. (2018b) also observed the anti-atrophic effect of quercetin in the obese mice. They found that quercetin inhibited the TNF-α-induced reduction of myotube diameter by downregulating both the mRNA and protein expression of MuRF1 and atrogin-1 in C2C12 myotubes. This effect was mediated by both inhibiting the NF-κB pathway and inducing heme oxygenase (HO)-1 which resulted from the activation of NRF-2 and. Similar results were obtained in an in vivo study which reported that quercetin supplementation decreased MuRF1 protein expression by downregulating NF-κB protein and increasing HO-1 and NRF-2 protein in the muscle tissues of high-fat diet-induced obese mice.

Notably, quercetin decreased plasma levels of signal transducer and activator of transcription 3 (STAT3) and IL-6 in *Apc*^Min/+^ mice, which is a model of cachexia with colorectal cancer (Moser et al., 1990), thus implying that quercetin inactivates the IL-6/STAT3 signaling pathway (Velázquez et al., 2014). Quercetin significantly increased body weight as well as the mass of the epididymal fat, gastrocnemius muscle, and quadriceps muscle. Additionally, when *Apc*^Min/+^ mice were supplemented with quercetin, there was an increase in the grip strength relative to the non-treated group. However, there was no difference in rotarod speed or run-to-fatigue performance. Trichostatin A is an anti-cancer drug with adverse effects, including heart hypertrophy, oxidative stress, and inflammation, which contributes to muscle wasting (Chan et al., 2018). In A549 lung cancer cell-bearing mice, quercetin enhances the anti-cancer activity of trichostatin A, as demonstrated by a decrease in the volume of tumors through the upregulation of p53. The reduced gastrocnemius muscle in trichostatin A-treated tumor-bearing mice was recovered by a quercetin-containing diet and an intraperitoneal injection of quercetin. At the molecular level, quercetin downregulated the protein expression of atrogin-1 and MuRF1 by keeping FoxO1 in cytosol. Moreover, lipid peroxidation and the levels of TNF-α and IL-1β in the plasma and the gastrocnemius muscle were also decreased by quercetin treatment due to its anti-oxidant and anti-inflammatory effects.

One study compared the efficacies of quercetin and flavone on disuse muscle atrophy with mice subjected to tail-suspension (Mukai et al., 2010). Flavone moderately recovered the weight of the gastrocnemius muscle; however, its effect was not significant and the degree of recovery did not reach the level observed in the quercetin-treated group. Furthermore, flavone treatment did not influence the expression of atrogin-1 and MuRF1. In contrast, the injection of quercetin into the gastrocnemius muscle increased its weight. Quercetin treatment significantly downregulated MuRF1 and atrogin-1 expression and reduced the thiobarbituric acid‐reactive substance (TBARS) content. Based on the results of this study, it is concluded that the hydroxyl groups in quercetin are important for attenuating tail-suspension-induced muscle atrophy. Similarly, quercetin prevented the development of denervation-induced muscle atrophy by increasing the weight of the gastrocnemius muscle (Mukai et al., 2016b). Although the study on muscle atrophy, which was induced by tail-suspension, found that quercetin downregulated MuRF1 (Mukai et al., 2010), quercetin did not affect the level of MuRF1 mRNA expression but significantly increased p-Akt, IGF-1, and PGC-1α in denervated mice (Mukai et al., 2016b). Notably, quercetin treatment stimulated the recovery of weight in damaged muscles and improved the intrinsic growth of neurons after the nerve-crush injury to the hindlimbs of mice (Chen et al., 2017).

The anti-atrophic effect of quercetin on muscle was also examined in animal models where atrophy was induced by dexamethasone or clinorotation (Hemdan et al., 2009). In C2C12 myotubes subjected to clinorotation, quercetin inhibited atrogin-1 and MuRF1 expression by downregulating the phosphorylation of ERK; however, quercetin did not reduce the expression of these genes in dexamethasone-treated C2C12 cells. The latter result is inconsistent with a previous study demonstrating that quercetin glycoside protected mice and C2C12 myotubes from dexamethasone-induced muscle atrophy (Otsuka et al., 2019). Quercetin glycoside decreased atrogin-1 and MuRF1 mRNA expression in dexamethasone-treated C2C12 myotubes. Quercetin glycoside increased the ratio of the gastrocnemius muscle to body weight in dexamethasone-treated mice by reducing atrogin-1, MuRF1, and myostatin mRNA expression. These discrepancies originate from differences in the models, used concentrations, or the structural differences between quercetin and quercetin glycosides. Under normal conditions, quercetin also has beneficial effects on muscle. Quercetin treatment stimulated mitochondrial biogenesis in muscle tissues and increased exercise tolerance via SIRT1 and PGC-1α in mice (Davis et al., 2009). In C2C12 myotubes, quercetin stimulated transcriptional activity at the Tfam promoter (Yoshino et al., 2015).

Several studies have examined the effect of quercetin on Duchenne muscular dystrophy (DMD) (Ballmann et al., 2015; Hollinger et al., 2015; Selsby et al., 2016; Spaulding et al., 2016). However, this effect is not discussed in this review, because DMD is a genetic disorder caused by a lack of dystrophin protein, but not by environmental factors.

### Isoflavan

#### Glabridin

Glabridin is a prenylated isoflavan derived from licorice, the root of liquorice (*Glycyrrhiza glabra*) (Wei et al., 2017). Ingestion of licorice flavonoid oil, which contained glabridin, significantly increased femoral muscle mass without affecting body weight in KK-A^y^ mice by decreasing the protein level of p-p38 and increasing the level of p-mTOR (Yoshioka et al., 2018). Based on a previous study, the effect of glabridin on muscle atrophy was also examined (Yoshioka et al., 2019). Glabridin prevented dexamethasone-induced muscle protein degradation in C2C12 myotubes and increased the weight of the tibialis anterior and gastrocnemius muscle tissues in mice. This study elucidated molecular mechanisms by which glabridin inhibited the loss of muscle protein and mass. Glabridin worked as an antagonist of the glucocorticoid receptor, indicating that glabridin competes with dexamethasone to bind to the glucocorticoid receptor. Subsequently, glabridin inhibited the translocation of the glucocorticoid receptor into the nucleus. Glabridin also inhibited the expression of MuRF1 and the RING-type ubiquitin ligase, Cbl-b, but failed to reduce atrogin-1 expression.

### Isoflavone

#### Corylifol A

Corylifol A is a geranylated isoflavone compound derived from babchi (*Psoralea corylifolia*) (Han et al., 2020). Under normal conditions, corylifol A increased both the transcriptional activity of MyoD and the protein expression of MyoD, myogenin, and MHC via p38 protein but not p-Akt, subsequently stimulating myogenesis. Likewise, corylifol A increased MHC and enhanced myogenesis in a dexamethasone-induced atrophic condition of C2C12 myotubes. Furthermore, corylifol A reduced NF-κB, MuRF1, atrogin-1, and myostatin in dexamethasone-treated C2C12 myotubes. However, to date, its anti-atrophic effect on muscle has not been demonstrated in the animal model.

#### Daidzein

Daidzein, with structural similarity to estrogen, is abundantly present in soybean (*Glycine max*) and exerts phytoestrogenic effects, particularly as an ERβ agonist (Ogawa et al., 2017). Hirasaka et al. (2013) reported that daidzein inhibited TNF-α-induced muscle atrophy by suppressing MuRF1 promoter activity in C2C12 myotubes. In TNF-α-stimulated C2C12 myotubes, the suppression of the promoter activity of MuRF1 in response to daidzein was primarily mediated by SIRT1 activation. Daidzein significantly increased the diameters of dexamethasone-treated C2C12 myotubes by upregulating the myogenic transcriptional factors, MyoD, myogenin, and MHC (Lee et al., 2017b). Furthermore, daidzein reduced LPS-induced expression of atrogin-1 in C2C12 myotubes (Shiota et al., 2015). Although three in vitro studies have directly examined the role of daidzein in muscle atrophy, some other studies have presented the positive effect of daidzein on the regulation of muscle function and physiology. Such studies can provide insights into the anti-atrophic effects of daidzein. Firstly, Zheng et al. (2018) described the anabolic effect of daidzein on muscle. This effect was demonstrated by increases in the number of C2C12 myotubes and the upregulation of MHC expression. Another study also showed the stimulatory effect of daidzein on myogenesis through MyoD, myogenin, and MHC, as well as its hypertrophic impact on muscle via the mTOR/p70S6K pathway (Lee et al., 2017b). Akt and p38 proteins were the primary mediators of these hypertrophic and myogenic effects of daidzein. However, daidzein did not influence protein synthesis and degradation in L6 myoblasts (Jones et al., 2005). The different results obtained from the C2C12 and L6 cells may be due to the origin of cell lines: C2C12 cells were derived from a mouse and L6 cells from a rat. Further, daidzein improved mitochondrial function, as demonstrated by increasing mtDNA content and upregulating OXPHOS-related genes (Yoshino et al., 2015). Regarding its effect on improving mitochondrial biogenesis, daidzein promoted Tfam expression through a SIRT1-mediated PGC-1α/NRF network. Finally, daidzein downregulated the expression of ubiquitin-specific peptidase 19 (USP19), which is a deubiquitinating enzyme, through ERβ-mediated transcription (Ogawa et al., 2017). A diet containing daidzein increased the soleus muscle mass of female mice but not of male mice by downregulating the USP19 protein.

#### Genistein

Genistein found in soybean (*G. max*) is an isoflavone and confers phytoestrogen activity (Hirasaka et al., 2013). Some studies have investigated the effects of genistein on muscle physiology, function, and signaling pathways using several models (Ding et al., 2017; Messina et al., 2011). Three studies have reported the anti-atrophic effect of genistein on muscle (Aoyama et al., 2016; Hirasaka et al., 2013; Shiota et al., 2015). An in vitro study reported that genistein significantly increased myotube diameter by downregulating MuRF1 promoter activity in TNF-α-treated C2C12 cells (Hirasaka et al., 2013). Genistein also downregulated atrogin-1 expression in LPS-treated C2C12 cells (Shiota et al., 2015). Consistent with the findings of these in vitro studies, in an animal study where muscle atrophy was induced by denervation, genistein increased the ratio of the soleus muscle to body weight via the ERα protein (Aoyama et al., 2016). Principally, ERα was activated in response to genistein, which subsequently inhibited the protein expression of FoxO1 and decreased atrogin-1 and MuRF1 mRNA expression. In *mdx* mice, a model for DMD, the anti-oxidant and anti-inflammatory effects of genistein were demonstrated by decreased NF-κB binding activity, TNF-α protein expression, and H_2_O_2_ content (Messina et al., 2011). Moreover, genistein increased the transcriptional activity at the Tfam promoter (Yoshino et al., 2015), the muscle diameter by upregulating IGF-1 and IGF-1R mRNA expression in C2C12 myotubes (Zheng et al., 2018), and protein synthesis in L6 myoblasts (Jones et al., 2005). Genistein also protected genioglossus myoblasts isolated from SD rats from hypoxia-induced oxidative stress and apoptosis via the PI3k/Akt and ERK pathways (Ding et al., 2017).

### Anthocyanidin

#### Delphinidin

Delphinidin is found in pigmented fruits and vegetables and confers a blue-red color (Murata et al., 2016). Two studies have suggested that delphinidin prevents muscle atrophy by characterizing its underlying mechanisms (Murata et al., 2016; 2017). Delphinidin significantly reversed MuRF1 expression by upregulating the nuclear factor of activated T cells 3 (NFATc3) and miR-23a (Murata et al., 2017). However, it did not affect the levels of p-Akt and p-FoxO3a. These results suggest that the reduction of MuRF1 expression in response to delphinidin treatment is mediated by the NFATc3/miR-23a pathway but not by the Akt/FoxO pathway. Delphinidin also reduced the expression of Cbl-b or a RING-type ubiquitin ligase in dexamethasone-treated C2C12 myotubes (Murata et al., 2016). In particular, Murata et al. (2017) compared cyanidin (3,3′,4′,5,7-pentahydroxyflavylium) and delphinidin (3,3′,4′,5,5′,7-hexahydroxyflavylium) to investigate their effects on MuRF1 expression. Immunoblot analysis and qRT-PCR results showed that cyanidin did not influence MuRF1 expression. This implies that the hydroxyl group at the 5′ site of anthocyanidin was crucial for regulating MuRF1 expression in dexamethasone-induced muscle atrophy. In the animal model, delphinidin prevented unloading-induced muscle atrophy in the gastrocnemius muscle by suppressing MuRF1 expression (Murata et al., 2017). A loss of quadriceps muscle mass in response to dexamethasone was also recovered by delphinidin administration, as observed by a reduction in Cbl-b and stress-related genes (Murata et al., 2016). Delphinidin also moderately decreased atrogin-1 mRNA expression in LPS-treated C2C12 myotubes, but the effect was not significant (Shiota et al., 2015). Overall, in terms of inhibiting muscle atrophy, delphinidin regulates MuRF1 and Cbl-b but not atrogin-1.

### Chalcone

#### 4-Hydroxyderricin

Recently, ashitaba (*Angelica keiskei*) extract has been reported to improve running performance and elevate the gastrocnemius muscle mass in dexamethasone-treated rats by decreasing MuRF1 and atrogin-1 expression and increasing MyoD and myogenin expression (Kweon et al., 2019). *A. keiskei* also stimulated myogenesis in C2C12 myotubes, as shown by an increase in MHC expression. Ten chalcones isolated from *A. keiskei* extract were effective in increasing the transcriptional activity of MyoD and MHC expression despite the degree of their effects. This result suggests that the anti-atrophic effect of *A. keiskei* extract on muscle may be attributable to a mixture of chalcone compounds. One compound, 4-hydroxyderricin, which represented 2.8% of an *A. keiskei* extract, had the most significant effect on MyoD transcription and MHC expression among the ten chalcone compounds. The chemical structures of 4-hydroxyderricin and isobavachalcone are similar, with the only difference being that the 7th position of 4-hydroxyderricin has a methoxy group and that of isobavachalcone has a hydroxyl group (Rong et al., 2014). In terms of the stimulatory effect of these compounds on MyoD transcriptional activity, 4-hydroxyderricin was more effective than isobavachalcone, indicating that the methoxy group in 4-hydroxyderricin is critical for stimulating myogenesis. 4-Hydroxyderricin also stimulated both myogenesis and myogenesis-related genes, including MHC, myogenin, and MyoD, by activating p38 protein (Kweon et al., 2019). Moreover, 4-hydroderricin treatment upregulated MHC expression and downregulated MuRF1, atrogin-1, and myostatin in three in vitro models of muscle wasting.

#### Isobavachalcone

Isobavachalcone is a prenylated chalcone compound found in ashitaba (*A. keiskei*) and babchi (*P. corylifolia*) (Han et al., 2020; Kweon et al., 2019). Isobavachalcone isolated from *A. keiskei* extract elevated both the transcriptional activity of MyoD and the protein expression of MHC in C2C12 myotubes (Kweon et al., 2019). This increase in the transcriptional activity of MyoD in response to isobavachalcone was consistent with the findings of another study in which isobavachalcone was derived from *P. corylifolia* (Han et al., 2020). Isobavachalone was reported to recover myogenesis in TNF-α-treated C2C12 cells by increasing the expression of MyoD, myogenin, and MHC (Hur et al., 2019). Isobavachalone was further found to affect other mechanisms associated with muscle atrophy: the inhibition of NF-κB nuclear translocation for an anti-inflammatory effect and the stimulation of NRF-2 translocation into nucleus for an antioxidant effect. In addition, isobavachalcone suppressed the expression of MuRF1 and atrogin-1 by reducing the level of p-FoxO1. However, demonstrations of its anti-atrophic effect were limited to in vitro experiments.

#### Panduratin A

Panduratin A is a prenylated chalcone compound isolated primarily from fingerroot (*Boesenbergia pandurata*) rhizomes (Kim et al., 2018a). In TNF-α-treated L6 myotubes, panduratin A increased the myotube diameter by activating the PI3K/Akt pathway (Sa et al., 2017). In particular, panduratin A increased the downstream factors, such as mTOR for protein synthesis, and myoD and myogenin for muscle differentiation. Conversely, it inhibited the nuclear translocation of FoxO3, preventing the autophagy-lysosomal system and ubiquitin E3 ligase expression. In addition, panduratin A reduced oxidative stress by lowering ROS and increasing the mRNA expression of the anti-oxidant enzymes, catalase and SOD. Previous studies revealed that panduratin A stimulated mitochondrial biogenesis by the PGC-1α signaling pathway in L6 myotubes and increased mtDNA content (Kim et al., 2016) and mitochondria-specific gene expression in the skeletal muscle of obese mice (Kim et al., 2011). Regulating and improving mitochondria function may be another mechanism by which panduratin A inhibited muscle atrophy; however, such a conclusion is not possible without additional data from muscle atrophy models.

## Future perspectives

The skeletal muscle is essential for the body function because it converts chemical energy into mechanical energy and metabolizes energy sources. Notably, the protein content in skeletal muscle is very critical because it is closely associated with muscle function. Muscle atrophy or muscle wasting is characterized by reductions in muscle mass and protein content. Chronic diseases (cachexia), aging (sarcopenia), and muscle disuse are the primary causes of muscle atrophy. Despite numerous attempts to develop agents and therapies to treat muscle atrophy, clinical applications are limited due to severe adverse effects and the lack of evidence for efficacy. Currently, more attention is directed towards the therapeutic potential of food ingredients, plant extracts, and phytochemicals for the treatment of muscle atrophy by focusing on their mechanisms of actions, such as myogenesis, protein turnover, and mitochondria function.

Flavonoids are bioactive polyphenol compounds found in food and plants. Flavonoids can be divided into several subgroups depending on their characteristics, such as oxidation, hydroxylation, and substitution. In addition, there are several derivative forms of flavonoids including aglycone and glycosylated, methylated, prenylated, and/or hydroxylated flavonoids. The biological activities exhibited by these diverse structures vary; certain activities are highly dependent on specific structures, variously affecting their potential activities against muscle atrophy. Additionally, for the inhibition of muscle atrophy, the molecular mechanisms of action in response to flavonoids are different as follows: protein turnover, mitochondrial activity, and myogenesis.

However, with reports from only two studies, there is insufficient information on the clinical applications of flavonoids against muscle atrophy. One study showed that baicalin reduced cachexia in cancer patients and the other reported that epicatechin increased grip strength in elderly participants. Although clinical tests of flavonoids to treat other diseases have been proposed, insufficient data still prevents the use of flavonoids in human subjects. However, published studies on flavonoids can help evaluate their anti-atrophic effects on muscle from the perspective of determining efficacies and safe concentration. Besides, their structural activities should be considered to identify candidates for human testing. Prenylation, methylation, and hydroxylation can modulate the degree of anti-atrophic effects. Finally, flavonoid-binding proteins that promote or antagonize muscle atrophy are mostly unknown. Collectively, although flavonoids have therapeutic potential against sarcopenia, cachexia, and disuse muscle atrophy, the effects of flavonoids on muscle atrophy for clinical application require further investigation.
