# Correction to: Flavonoids: nutraceutical potential for counteracting muscle atrophy

**DOI:** 10.1007/s10068-020-00846-z

**Published:** 2020-11-20

**Authors:** Changhee Kim, Jae-Kwan Hwang

**Affiliations:** Department of Biotechnology, Yonsei University, 50 Yonseiro, Seodaemun-gu, Seoul, 03722 Republic of Korea

## Correction to: Food Sci Biotechnol 10.1007/s10068-020-00816-5

The article “Flavonoids: nutraceutical potential for counteracting muscle atrophy”, written by Changhee Kim, Jae-Kwan Hwang was originally published electronically on the publisher’s internet portal on 16 September 2020 without open access. With the author(s)’ decision to opt for Open Choice the copyright of the article changed on 23 October 2020 to © The Author(s) 2020 and the article is forthwith distributed under a Creative Commons Attribution 4.0 International License, which permits use, sharing, adaptation, distribution and reproduction in any medium or format, as long as you give appropriate credit to the original author(s) and the source, provide a link to the Creative Commons license, and indicate if changes were made. The images or other third party material in this article are included in the article’s Creative Commons license, unless indicated otherwise in a credit line to the material. If material is not included in the article’s Creative Commons license and your intended use is not permitted by statutory regulation or exceeds the permitted use, you will need to obtain permission directly from the copyright holder. To view a copy of this license, visit http://creativecommons.org/licenses/by/4.0.

The original article has been corrected.

